# Single‐Cell Transcriptomics Identifies BST2 as an Oncogenic Driver and Immunotherapy Biomarker in Lung Adenocarcinoma

**DOI:** 10.1155/mi/3777132

**Published:** 2026-05-06

**Authors:** Keyun Zhu, Mingrong Lin, Chengbin Lin, Tao Hu, Zhikai Cao, Shuo Huang, Yan Shen, Jing Zeng, Jinxian He, Weiyu Shen, Jianjian Ying

**Affiliations:** ^1^ Department of Thoracic Surgery, The Affiliated LiHuiLi Hospital of Ningbo University, Ningbo, 315040, Zhejiang, China, nbu.edu.cn

**Keywords:** immune cells, LUAD, TME

## Abstract

**Background:**

Lung adenocarcinoma (LUAD) is a leading cause of cancer‐related mortality worldwide. The tumor microenvironment (TME) plays a pivotal role in LUAD progression, but the specific molecular mechanisms driving malignancy and immune evasion remain incompletely understood. Bone marrow stromal cell antigen 2 (BST2) has been implicated in other cancers, yet its functional role and therapeutic potential in LUAD require further elucidation.

**Methods:**

We integrated single‐cell RNA sequencing (scRNA‐seq) data from primary LUAD tissues and normal lung tissues with bulk RNA‐seq data from The Cancer Genome Atlas (TCGA)–LUAD and GEO cohorts. Malignant epithelial cells were identified using inferCNV analysis. Cellular trajectory and cell–cell communication analyses were systematically performed to delineate the malignant transformation process and its interactions with the TME. High‐dimensional weighted gene coexpression network analysis further identified key functional modules within malignant subpopulations. The oncogenic role of BST2 was comprehensively validated through differential expression analysis, survival analysis, gene set enrichment analysis (GSEA), and in vitro cellular assays, including RT‑qPCR, MTT, colony formation, Transwell invasion, and wound healing experiments. In addition, we employed the tumor immune dysfunction and exclusion (TIDE) algorithm to evaluate its association with immunotherapy response and performed drug screening via the Clue.io platform to explore its therapeutic potential.

**Results:**

scRNA‐seq analysis revealed significant heterogeneity in the TME and identified two distinct subpopulations of malignant epithelial cells. Trajectory analysis uncovered a specific lineage (Lineage 4) driving the normal‐to‐malignant transition, while cell communication analysis highlighted interactions between malignant cells and tumor‐associated macrophages (TAMs) mediated by MIF and APP signaling pathways. High‐dimensional weighted gene coexpression network analysis (hdWGCNA) identified a coexpression gene module (Module 1) specifically enriched in malignant subpopulations. By intersecting with a set of immunoregulatory genes, BST2 was ultimately determined as a key candidate oncogene. BST2 was significantly upregulated in malignant epithelial cells and TAMs, and its high expression was closely associated with poor patient prognosis. GSEA demonstrated that high BST2 expression was linked to the activation of crucial oncogenic pathways, including oxidative phosphorylation, Kras signaling, and epithelial–mesenchymal transition (EMT). In vitro validation further confirmed that BST2 knockdown suppressed LUAD cell proliferation, migration, and invasion. Furthermore, elevated BST2 expression was associated with reduced efficacy of immune checkpoint blockade (ICB) therapy.

**Conclusion:**

Our study unveils BST2 as a critical oncogene in LUAD, promoting tumor progression and influencing the TME, particularly via TAM recruitment. BST2 expression predicts patient prognosis and immunotherapy response, positioning it as a promising biomarker and therapeutic target.

## 1. Introduction

Lung cancer remains the leading cause of cancer‐related mortality worldwide [1], with lung adenocarcinoma (LUAD) representing its most prevalent histological subtype that has drawn significant research interest [2]. Recent years have witnessed remarkable progress in multimodal treatment strategies, including targeted therapy, immunotherapy, radiotherapy, and noninvasive surgical resection [3]. With the successive approval and clinical application of novel targeted agents such as the PD‐1 inhibitor pembrolizumab and the EGFR inhibitor furmonertinib [4], the 5‐year overall survival (OS) rate has improved for LUAD patients with corresponding targetable biomarkers. Nevertheless, the overall 5‐year OS for LUAD patients remains below 20%, underscoring the ongoing severity of the therapeutic challenge [5]. Consequently, exploring novel therapeutic targets and expanding precision treatment avenues have become crucial for improving outcomes in LUAD patients [6].

The tumor microenvironment (TME) refers to the complex and highly dynamic multicellular milieu in which tumor cells reside, playing a central role in key biological behaviors such as tumor proliferation, migration, and apoptosis [7]. The TME responds to both endogenous and exogenous stresses, various stimuli, and therapeutic interventions, ultimately promoting cancer cell survival and dissemination. This microenvironment encompasses a variety of immune cells, including B cells, T cells, dendritic cells (DCs), tumor‐associated macrophages (TAMs) [8], and natural killer (NK) cells, as well as multiple stromal components such as cancer‐associated fibroblasts (CAFs), endothelial cells (ECs), and smooth muscle cells (SMCs) [9]. Furthermore, the TME includes the extracellular matrix (ECM), diverse cell–secreted factors, and the tumor‐associated vasculature. A well‐established mechanism by which the TME mediates tumor immune escape is the PD‐L1/PD‐1 pathway: Both cancer cells and TAMs frequently overexpress the immune checkpoint protein PD‐L1, which binds to the PD‐1 receptor on adaptive immune cells, leading to T cell dysfunction [10] and impaired immune surveillance [10–12]. Based on this mechanism, inhibiting the PD‐L1/PD‐1 axis through immune checkpoint blockade (ICB) has increasingly become a standard treatment for a wide range of cancers [13].

Bone marrow stromal cell antigen 2 (BST2), also known as HM1.24 or CD317, is a type II transmembrane protein pivotal in antiretroviral defense within innate immunity [14]. Clinically, elevated BST2 expression correlates significantly with Braf mutations, dMMR/MSI‐H status, and immune activation, positioning it as a potential biomarker for immunotherapy response. Its oncogenic role has been established in several cancers, including multiple myeloma [15], breast cancer [16], and renal carcinoma [17]. Although BST2 expression has been observed in lung cancer cell lines and LUAD tissues [18], its specific oncogenic functions in LUAD require further independent validation.

The advancement of bioinformatics has provided powerful support for identifying critical therapeutic targets and novel predictive biomarkers [19].

This study utilized single‐cell RNA sequencing (scRNA‐seq) to systematically compare the TME heterogeneity between normal lung tissues and primary LUAD. Our analysis identified a distinct subpopulation of tumor cells exhibiting high BST2 expression, which was closely associated with tumorigenesis and progression. Simultaneously, BST2 was found to be highly enriched in a subpopulation of TAMs, suggesting its potential role in influencing LUAD progression through the modulation of the TAM function. And that TAMs are indispensable components of the TME and actively promote tumor development. We further validated through in vitro experiments that BST2 indeed drives malignant tumor phenotypes. In conclusion, this study not only reveals a novel BST2‐mediated mechanism underlying LUAD progression but also proposes it as a promising candidate target for precision therapy.

## 2. Material and Methods

### 2.1. Collection and Preprocessing of Data

This study included a total of 865 samples: four scRNA‐seq samples (two primary LUAD and two normal lung tissues) from GEO datasets GSE235782 and GSE289881, 439 LUAD bulk RNA‐seq samples from GSE68465, and 422 RNA‐seq samples from the The Cancer Genome Atlas (TCGA)–LUAD cohort (https://portal.gdc.cancer.gov/). Gene expression data from TCGA–LUAD were retrieved using the TCGAbiolinks R package, converted to TPM format, and log2‐transformed for normalization.

scRNA‐seq data quality control and downstream analyses were conducted using Seurat (v4.3.0). Cells were filtered based on the following criteria: UMI counts between 200 and 7000 and mitochondrial gene expression ≤20%. The DoubletFinder package (v2.0.3) was employed to remove potential doublets. Following data normalization using the NormalizeData function, dimensionality reduction was performed through principal component analysis (PCA). The RunUMAP function was subsequently applied for further dimensionality reduction. Cell clustering was conducted using the FindNeighbors and FindClusters functions (dims = 1:30 and resolution = 0.2), and batch effects were corrected using the Harmony R package. Cell types were annotated using marker genes from the CellMarker database [20].

### 2.2. Cell Trajectory and Developmental Analysis

Cell trajectory analysis was performed using the SCOP package (v0.5.3), and the slingshot algorithm was applied to infer dynamic gene expression changes and biological functional evolution between normal epithelial cells and malignant epithelial cells.

### 2.3. Cell–Cell Communication

The cell–cell crosstalk between malignant epithelial cells and other cell types was calculated by the “CellChat” [21] package (v1.6.1).

### 2.4. CNV and Downstream Single‐Cell Analysis

Epithelial cell expression matrices from two LUAD samples (T1 and T2) in the GSE235782 dataset were extracted. To identify copy number variations (CNVs) in malignant epithelial cells of LUAD, we employed the “inferCNV” R package. Malignant lung epithelial cells were identified using a CNV correlation > 0.3 and a CNV score > 0.002. The “hdWGCNA” R package was utilized to identify coexpressed gene modules in tumor subpopulations [22]. Specifically, based on gene expression variance across all malignant epithelial cells from LUAD scRNA‐seq profiles, a soft threshold power of six was selected, and genes were clustered into four distinct modules.

### 2.5. Gene Set Enrichment Analysis (GSEA)

Based on BST2 gene expression levels, TCGA–LUAD samples were stratified into high‐ and low‐expression groups. GSEA of differentially expressed genes between these two groups was performed using the gseKEGG function from the clusterProfiler R package, with reference to the Molecular Signatures Database (c2.cp.kegg.v2025.1.Hs.symbols.gmt). A false discovery rate (FDR) < 0.05 was set as the significance threshold, and the 17 pathways with the highest enrichment scores were selected for visualization.

### 2.6. Cell Culture, RT‐qPCR, Transfection, and Cell Function Assays

Five LUAD cell lines (A549, H1299, PC‐9, H1975, and HCC827) and the normal lung cell line (BEAS‐2B) were obtained from the Type Culture Collection of the Chinese Academy of Sciences. In a 5% CO2 and humidified atmosphere at 37°C, the cells were cultured in RPMI‐1640 medium (Gibco, USA) containing 1% penicillin‐streptomycin (PS; HyClone) and 10% fetal bovine serum (FBS; PAN‐Seratech). The primer sequences are provided in the Supporting Information 1: Table S1. The siRNAs of BST2 constructs were synthesized by Tsingke Biotech (Tianjin, China), and sequences are listed in Supporting Information 1: Table S2. The interference efficiencies of si‐BST2‐1, si‐BST2‐2, and si‐BST2‐3 were 0.741, 0.684, and 0.458, respectively. The protocol of RT‐qPCR analysis was conducted as previously described [23]. The protocol of cell transfection was conducted as previously described [24]. The MTT assay and colony formation assay were used to assess cell proliferation ability. Transwell assays (with or without Matrigel) and wound healing assays were used to assess cell invasion and migration. All the experiments were performed as previously described [23].

### 2.7. Immunohistochemistry (IHC)

Tumor tissues embedded in paraffin were sectioned, deparaffinized, and rehydrated through a graded ethanol series. Antigen retrieval was carried out in citrate buffer, and the endogenous peroxidase activity was blocked. Sections were incubated with BST2‐specific primary antibodies (ID: 13560‐1‐AP, Proteintech, China) overnight at 4°C, followed by incubation with HRP‐conjugated secondary antibodies. Signal detection was performed using DAB, and the nuclei were counterstained with hematoxylin. Stained slides were examined under an Olympus microscope, and ImageJ software was applied for quantitative analysis.

### 2.8. Statistical Analysis

Data from at least three independent experiments are expressed as the mean ± standard deviation (SD). Statistical comparisons between groups were performed using a two‐tailed Student’s *t*‐test. All analyses and graphical representations were conducted with GraphPad Prism software (version 8.0, GraphPad Software, CA). A *p*‐value of less than 0.05 was considered statistically significant.

## 3. Results

### 3.1. Identification of Different Cell Types in LUAD by scRNA‐seq

**Figure 1:**
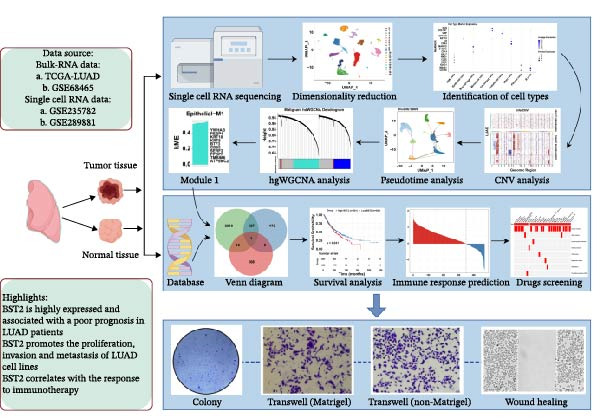
Flowchart of the study design. LUAD, lung adenocarcinoma; BST2, bone marrow stromal cell antigen 2; TCGA, The Cancer Genome Atlas; hdWGCNA, high‐dimensional weighted gene coexpression network analysis.

**Figure 2:**
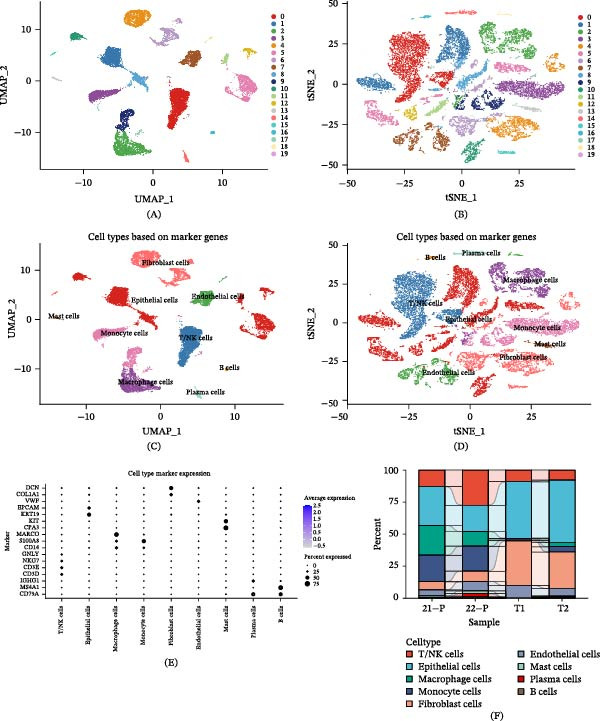
Identification of different cell types in LUAD by single‐cell RNA sequencing. (A, B) Visualization of 20 cell clusters identified in LUAD samples using UMAP and t‐SNE dimensionality reduction, respectively. (C, D) Annotation of cell clusters into nine cell types based on marker genes, displayed via UMAP and t‐SNE, respectively. (E) Dot plot showing expression levels of characteristic marker genes used to define the nine cell types. (F) Proportions of the nine cell types in LUAD samples.

The overall flow chart of our study is illustrated in Figure 1. Using the Seurat pipeline for quality control, we obtained a total of 29,225 high‐quality cells, including 19,052 cells from primary LUAD samples (T1 and T2) and 10,173 cells from normal lung tissues (21P and 22P). Based on PCA, all cells were clustered into 20 distinct subpopulations at a resolution of 0.2. Both Uniform Manifold Approximation and Projection (UMAP, Figure 2A) and t‐distributed stochastic neighbor embedding (t‐SNE, Figure 2B) plots showed clear spatial separation among the distinct cell clusters. Through cell annotation, we successfully identified nine major cell types: B cells, plasma cells, mast cells, epithelial cells, fibroblast cells, monocyte cells, ECs, T/NK cells, and macrophage cells. Their spatial distributions are shown in the UMAP (Figure 2C) and t‐SNE (Figure 2D) plots. Cell markers used for annotation were sourced from the CellMarker database (Figure 2E). This analytical approach effectively distinguished different cell types; for instance, using EPCAM and KRT19 as epithelial cell markers, we identified seven cell clusters (1, 5, 8, 10, 11, 12, and 19) as epithelial cells. Figure 2F further illustrates the proportional distribution of these nine cell types across all LUAD samples. In summary, this study successfully established a single‐cell transcriptomic atlas of LUAD and systematically identified nine major cell types, providing a foundation for further mechanistic investigations.

### 3.2. Malignant Cells in LUAD Are Identified by inferCNV Analysis

**Figure 3:**
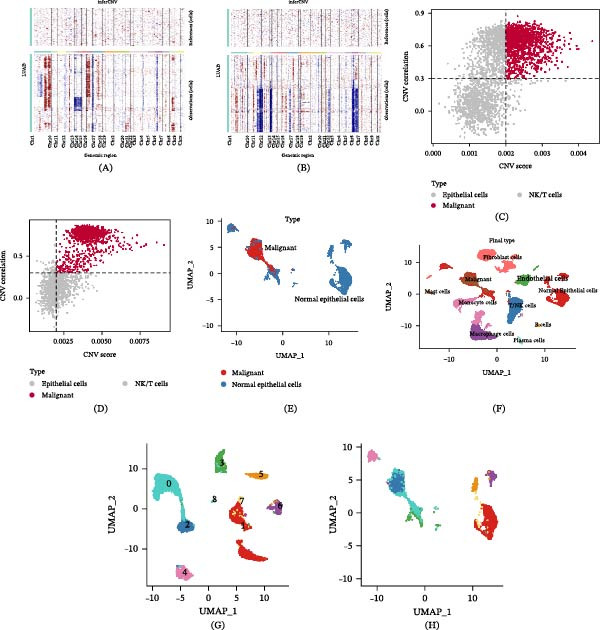
Identification of malignant cells in LUAD via inferCNV analysis. (A, B) CNV heatmaps showing whole‐chromosome copy number variations in epithelial cells of T1 and T2 samples, respectively. (C, D) Scatter plots displaying the CNV correlation and CNV scores of LUAD cells in T1 and T2 samples. (E, H) Distribution of normal epithelial cells and malignant epithelial cells in the UMAP visualization. (F) Distribution of malignant epithelial cells among all cells in the UMAP visualization. (G) UMAP plot showing nine epithelial subclusters identified through reclustering.

Distinguishing between benign and malignant epithelial cells at the single‐cell level using inherent cell markers presents significant limitations. To address this, we employed the inferCNV algorithm to assist in identifying malignant cells. First, using CNV of T/NK cells as a reference background, we inferred the CNV status of epithelial cells. As shown in Figure 3A,B, epithelial cells from both T1 and T2 samples exhibited significant CNV amplifications or deletions. Malignant epithelial cells were then accurately isolated based on CNV correlation and CNV scoring. Figure 3C,D demonstrates that the malignant cells defined by inferCNV showed high heterogeneity in gene expression, clearly distinguishing them from the control T/NK cells and normal epithelial cells. Through this analytical approach, we identified a total of 2612 confirmed malignant epithelial cells. Further dimensionality reduction and clustering analysis (resolution set to 0.1) of these cells revealed two distinct malignant epithelial subpopulations (Figure 3E,H). For integrated analysis, these malignant epithelial subpopulations were reincorporated into the overall cellular atlas (Figure 3F). Notably, in the reclustered epithelial cells, clusters 0 and 2 were identified as the malignant epithelial cell populations (Figure 3G). This study successfully established a workflow for identifying malignant cells in LUAD, providing a reliable foundation for further in‐depth investigations.

### 3.3. Malignant Transition of Epithelial Cells in LUAD Is Driven by the TME

In LUAD, the differentiation process from normal epithelial cells to tumor epithelial cells is a critical aspect of studying tumor progression. Through cell trajectory analysis, we identified five potential differentiation lineages among epithelial cells (Figure 4A,B), among which Lineage 4 specifically represents the transition from normal to malignant epithelial cells (Figure 4C). Further analysis of dynamic gene expression along this trajectory revealed that GADD45G, DDIT4, CPB1, and IFI6 were specifically highly expressed in malignant epithelial cells (Figure 4D). Notably, IFI6 [25] and CPB1 [26] have been previously reported to participate in tumor progression. These findings collectively elucidate the key molecular pathways involved in the malignant transformation of epithelial cells in LUAD.

Figure 4LUAD’s TME influences malignant epithelial cell transition. (A–C) The trajectory of normal epithelial cells evolving into malignant epithelial cells in LUAD was revealed, visualized through the distribution of two cell states (A), pseudotime lineage analysis (B), and five distinct evolutionary paths (C). (D) Heatmap showing the smoothed expression values of the top 20 genes with the most significant expression changes during the evolution along Lineage 4. (E, F) Interaction networks between malignant epithelial cells and other cell types, displaying both the number and strength of interactions. (G, H) Highly correlated signaling pathway networks between malignant epithelial cells and macrophages, specifically highlighting the MIF and APP signaling pathways. (I, J) Dot plots illustrate the interactions between malignant epithelial cells and macrophages, depicting malignant epithelial cells as signal senders (I) and signal receivers (J), respectively.

**Figure:**
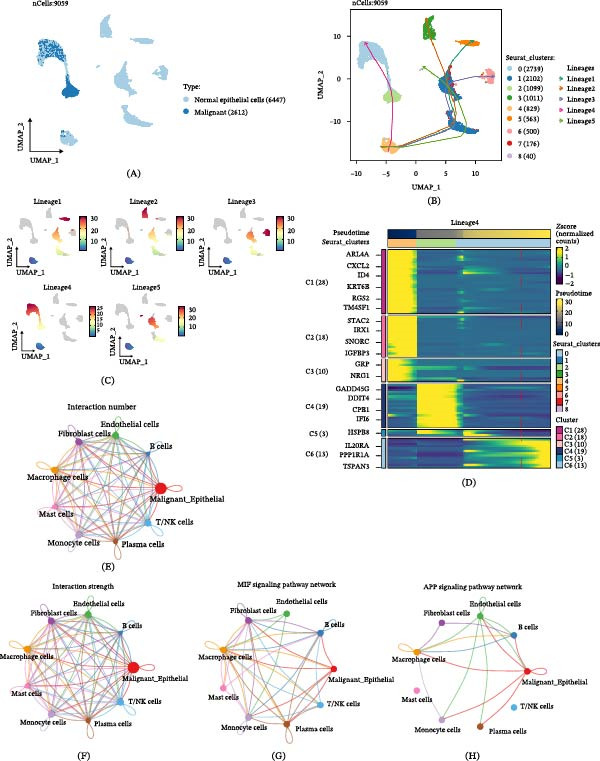

**Figure:**
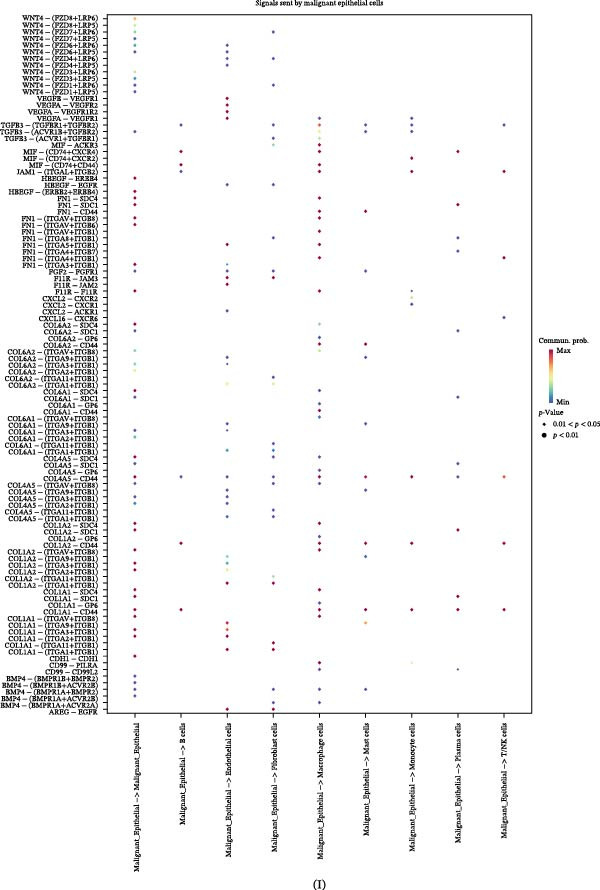

**Figure:**
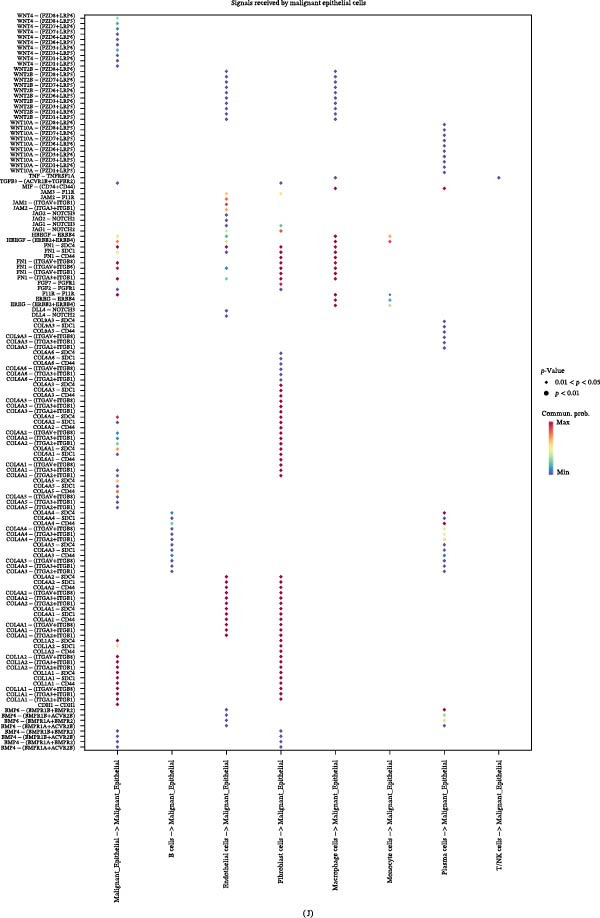

Cellular signaling and communication within the TME are crucial for cancer progression and metastasis [27]. CellChat analysis of the number and strength of cellular interactions revealed that the communication between malignant epithelial cells and TAMs was significantly stronger than that with other cell types (Figure 4E,F). Further mechanistic investigation indicated that malignant epithelial cells may actively recruit TAMs through the MIF and APP signaling pathways (Figure 4G,H) [28, 29]. A reciprocal communication between malignant epithelial cells and TAMs, mediated by FN1 and MIF, drives protumorigenic signaling (Figure 4I,J). This discovery clarifies the key mechanism by which malignant epithelial cells influence the tumor immune microenvironment by regulating TAMs via specific signaling molecules.

### 3.4. Gene Coexpression Modules in LUAD Cells Were Identified

Given that Subpopulations 0 and 2, as malignant epithelial cell clusters, are closely associated with poor prognosis in LUAD, it is essential to investigate the coexpressed gene networks playing critical roles in these two subpopulations. We constructed scale‐free coexpression networks for Subpopulations 0 and 2, achieving optimal network connectivity with a soft threshold set at six (Figure 5A,B). Through high‐dimensional weighted gene coexpression network analysis (hdWGCNA) analysis, four stable coexpression modules were identified, with the top 10 representative genes for each module displayed in Figure 5C,D. Module correlation analysis revealed that Module 1 and Module 3 showed significant correlation with each other, while Module 2 and Module 4 formed another distinct correlated module group (Figure 5E). Notably, the enrichment scores of Module 1 were specifically concentrated in Subpopulations 0 and 2, with minimal enrichment observed in other cell subpopulations (Figure 5F). Consistently, harmonization module characteristic genes (hME) of Module 1 were significantly increased in tumor’s sample and malignant epithelial cells (Figure 5G,H). These findings indicate that the genes in Module 1 constitute a unique coexpression network characteristic of Subpopulations 0 and 2, which may play an important role in the malignant progression of LUAD.

Figure 5Gene coexpression modules in LUAD cells were identified. (A) High‐dimensional weighted gene coexpression network analysis (hdWGCNA) was constructed based on the gene expression profiles of malignant epithelial cells. (B) Dendrogram displaying the four core gene modules identified by hdWGCNA. (C) Top 10 hub genes in each module selected based on intramodular connectivity ranking. (D) UMAP visualization showing the overall expression distribution of each gene module across epithelial cells. (E) Heatmap illustrating the expression correlations among the four gene modules. (F) Specific expression patterns of Module 1 across nine epithelial cell subpopulations. (G) Differential analysis of Module 1 expression scores among all samples. (H) Comparison of Module 1 expression between malignant epithelial cells and normal epithelial cells.

**Figure:**
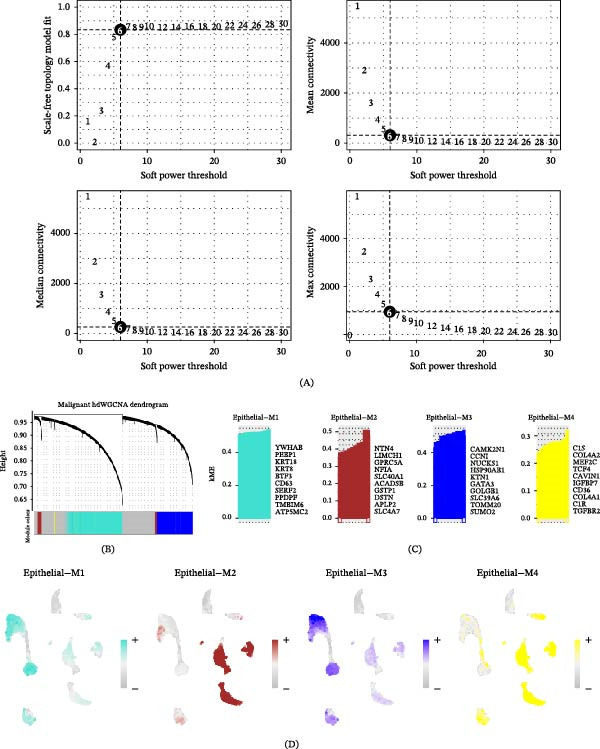

**Figure:**
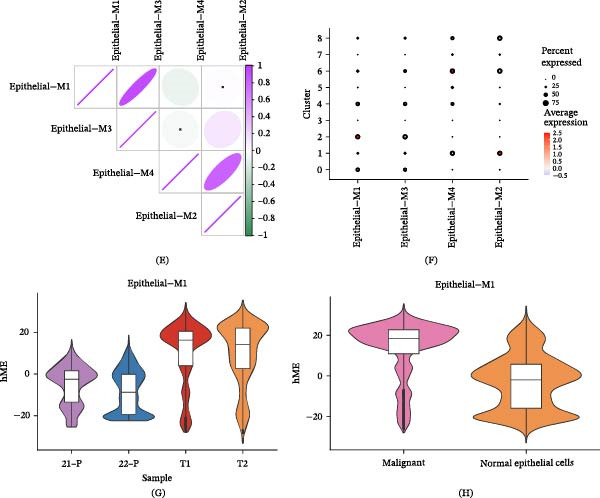

### 3.5. BST2 Was Identified as an Oncogene

**Figure 6:**
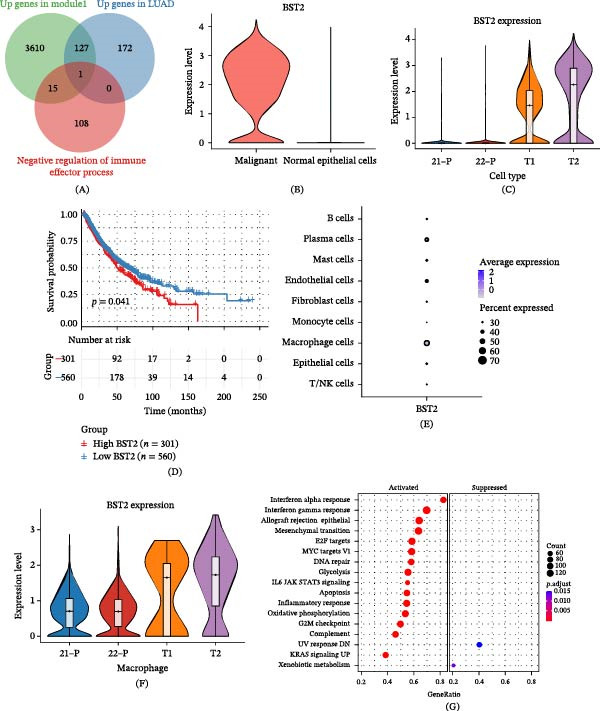
BST2 was identified as an oncogene. (A) Venn diagram showing the intersection of genes from Module 1, upregulated genes in malignant epithelial cells, and immune‐negative regulatory genes. (B) Expression levels of the BST2 gene in malignant epithelial cells versus normal epithelial cells. (C) Comparison of BST2 expression scores between malignant tissues and matched normal tissues. (D) Survival analysis of LUAD patients stratified by high and low BST2 expression levels. (E) Expression distribution pattern of BST2 across different cell types in LUAD. (F) Differential expression of BST2 in macrophage subpopulations between malignant and normal tissues. (G) GSEA pathway enrichment analysis of differentially expressed genes between high and low BST2 expression groups in LUAD.

To uncover key genes driving tumorigenesis, we performed differential expression analysis between normal lung tissues and LUAD tissues, identifying 300 genes significantly upregulated in tumor tissues (Supporting Information 2: Table S3). By intersecting this gene set with the Module 1 gene set (Supporting Information 3: Table S4) and the “negative regulation of immune effector process” gene set, BST2 was determined as the sole candidate gene (Figure 6A). To validate its transcriptional level, single‐cell transcriptomic analysis demonstrated that BST2 was specifically highly expressed in malignant epithelial cells and primary LUAD tissues (Figure 6B,C). Survival analysis further revealed that high BST2 expression was significantly associated with poor prognosis in LUAD patients (Figure 6D). Additionally, BST2 showed relatively high expression in macrophages and ECs (Figure 6E), with particularly prominent expression in tumor‐infiltrating macrophages (Figure 6F). GSEA enrichment analysis revealed that BST2 high‐expression–related genes were significantly enriched in multiple core tumor pathways, including oxidative phosphorylation, KRAS signaling pathway upregulation, and epithelial–mesenchymal transition (EMT) (Figure 6G). These results suggest that BST2 may contribute to disease progression in LUAD by synergistically driving these key processes. In summary, this study confirms that BST2 is significantly upregulated in LUAD, and its high expression is closely related to tumor progression and unfavorable patient prognosis.

### 3.6. Individualized Therapy for LUAD Patients Based on BST2

**Figure 7:**
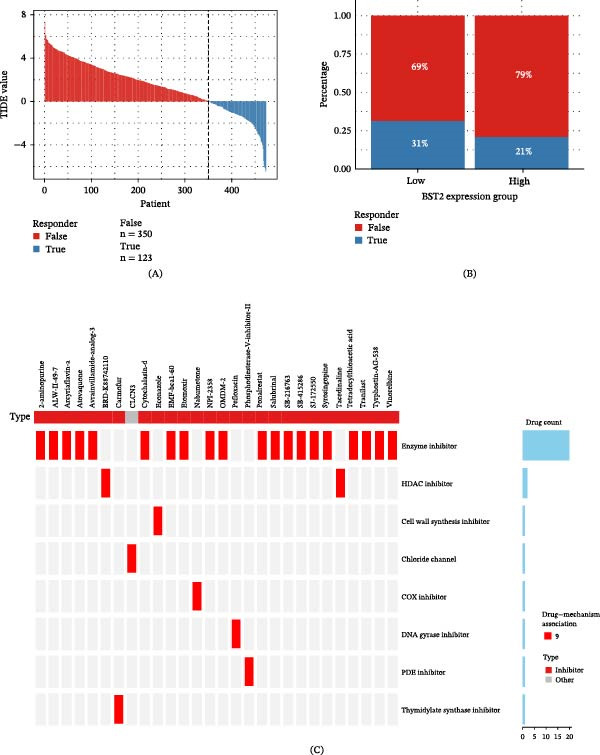
Individualized therapy for LUAD patients based on BST2. (A) Correlation analysis between TIDE scores and patient response status to immunotherapy. (B) Comparison of clinical response status to immunotherapy between patients with high and low BST2 expression levels. (C) Potential targeted therapeutic candidates screened based on high BST2 expression profiles.

The therapeutic options for LUAD remain limited, underscoring the urgent need to develop novel and effective therapeutic targets. In this study, we standardized the gene expression matrix from the TCGA dataset and employed the tumor immune dysfunction and exclusion (TIDE) algorithm to predict responses to immune checkpoint inhibitor therapy. As shown in Figure 7A,B, a low TIDE score was associated with a favorable response to immune checkpoint inhibitors, whereas high BST2 expression correlated with diminished therapeutic efficacy, suggesting BST2 as a potential predictor of the ICI response.

Furthermore, we screened for potential therapeutic agents targeting BST2‐high phenotypes using the Clue.io platform (Supporting Information 4: Table S5). Our analysis identified 28 compounds acting through eight molecular pathways in the high‐BST2 group (Figure 7C), with the most prevalent mechanisms of action being enzyme inhibition and HDAC inhibition. Previous studies have validated the efficacy of these inhibitory approaches in cancer treatment [30].

### 3.7. Downregulation of BST2 Inhibits LUAD Progression

**Figure 8:**
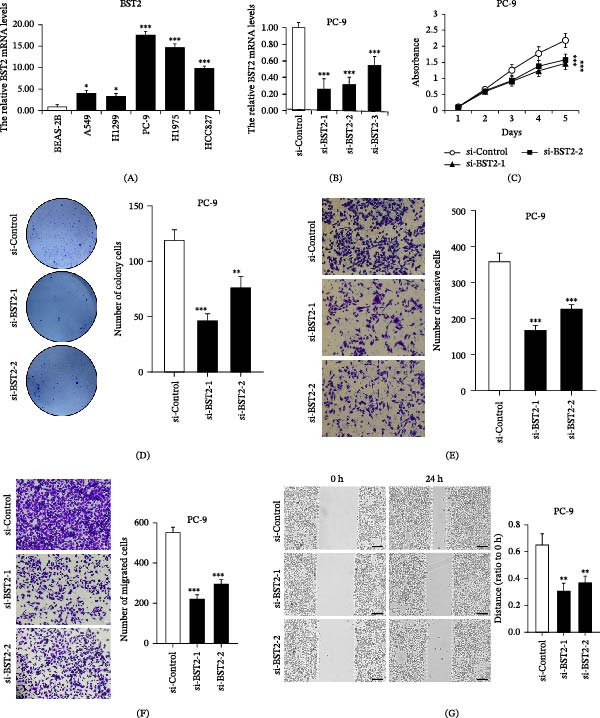
Downregulation of BST2 inhibits LUAD progression in PC‐9 cells. (A) The relative BST2 mRNA levels in lung cancer cells and normal lung epithelial cell line detected by RT‐qPCR. (B) Verification of BST2 knockdown efficiency by RT‐qPCR in PC‐9 cells. (C) The cell proliferation of BST2‐deleted PC‐9 cells detected by MTT assay. (D) Colony formation assay in BST2‐deleted PC‐9 cells. The cell invasion and migration assay of BST2‐deleted PC‐9 cells with (E) or without Matrigel (F). (G) Wound healing assays of BST2‐deleted PC‐9 cells.  ^∗^ *p* < 0.05,  ^∗∗^ *p*  < 0.01, and  ^∗∗∗^ *p*  < 0.001.

To investigate BST2 expression in LUAD, we measured BST2 mRNA expression levels in a panel of LUAD cell lines (A549, H1299, PC‐9, H1975, and HCC827) using the normal lung epithelial cell line BEAS‑2B as a control. RT‑qPCR revealed that BST2 mRNA expression was significantly higher in cancer cells compared to normal lung epithelial cells, with PC‑9 cells showing the highest expression level (Figure 8A). Meanwhile, we obtained paired tumor and adjacent normal tissue samples from 20 LUAD patients and performed immunohistochemical staining to investigate BST2 expression levels. The results align with our cellular findings, demonstrating a significant upregulation of BST2 in LUAD tissues (Supporting Information 5: Figure S1A). To further explore the biological function of BST2 in LUAD, we established PC‑9 cells with transient siRNA‑mediated BST2 knockdown. RT‑qPCR confirmed efficient knockdown of the target gene, and the si‑BST2‑1 and si‑BST2‑2 constructs exhibiting the strongest inhibitory effect were selected for subsequent functional studies (Figure 8B). In terms of cell proliferation, MTT assays demonstrated that BST2 knockdown significantly suppressed the proliferative activity of PC‑9 cells (Figure 8C). Consistently, colony formation assays further confirmed the inhibitory effect of BST2 downregulation, showing a reduction in both the quantity and dimensions of cell colonies (Figure 8D). Regarding metastatic ability, Transwell assays (with or without Matrigel) consistently showed that BST2 knockdown effectively inhibited cell invasion (Figure 8E) and migration (Figure 8F), while wound‑healing assays also confirmed impaired migratory capacity (Figure 8G). Consistent results were also validated in the H1975 cell line (Supporting Information 5: Figure S1B–F). In summary, our multidimensional functional experiments demonstrate that BST2 knockdown significantly suppresses the proliferation, colony formation, invasion, and migration of LUAD cells, suggesting that BST2 plays a protumorigenic role in lung cancer progression and holds potential as a therapeutic target.

## 4. Discussion

This study systematically elucidates the central role of BST2 in the malignant progression and immune microenvironment regulation of LUAD through integrated multiomics analysis. Based on single‐cell transcriptomic analysis, we revealed the high heterogeneity of the TME in LUAD and identified malignant epithelial subpopulations associated with specific CNVs. Trajectory analysis further delineated a specific lineage (Lineage 4), capturing the critical process of normal‐to‐malignant transformation. Genes dynamically upregulated along this trajectory (such as IFI6 and CPB1) highlight their potential roles in promoting the malignant phenotype, consistent with previous findings in other cancers [21, 22]. Cell–cell communication analysis suggests that interactions between malignant epithelial cells and TAMs, mediated by signals such as MIF, are particularly critical, indicating that tumor cells actively modulate the immune microenvironment to support their own growth and immune evasion [31–33].

Against this backdrop, hdWGCNA analysis focused on the coexpression network within malignant epithelial subpopulations, screening key modules associated with immune regulation and ultimately identifying BST2 as the core candidate gene. Experimental validation demonstrated that BST2 is significantly overexpressed in LUAD tissues and cell lines, and its expression level is closely associated with poor patient prognosis. Functionally, high BST2 expression is enriched in pro‐oncogenic pathways such as oxidative phosphorylation, KRAS signaling [34, 35], and EMT [36, 37]. Knockdown experiments further confirmed that silencing BST2 significantly inhibits the proliferation, migration, and invasive capabilities of tumor cells, providing multifaceted support for its role as a key driver of LUAD progression.

The innovation of this study lies in its use of an integrated multiomics strategy to position BST2 at the intersection of malignant transformation and immune regulation in LUAD. This elucidates its role in the intrinsic malignant behavior of tumor cells and reveals its close association with TAM recruitment and the formation of an immunosuppressive microenvironment. Of greater clinical significance, predictions based on the TIDE algorithm suggest that high BST2 expression may correlate with poor response to immune checkpoint inhibitor therapy, providing a clue for its potential as a novel biomarker for predicting immunotherapy resistance. Furthermore, using the Clue.io platform, we screened potential therapeutic agents targeting the high BST2 expression phenotype, offering a translational direction for future strategies combining BST2‐targeted therapies.

Nevertheless, this research has certain limitations, including the need for experimental validation of computational predictions and the potential impact of limited sample size on the generalizability of conclusions. Future research should involve larger‐scale prospective cohorts and mechanistic experiments to deepen the understanding of BST2’s functional network within the LUAD microenvironment and its clinical value, thereby advancing its translation into precision diagnostic and therapeutic targets.

## 5. Conclusion

In summary, our integrated analysis reveals BST2 as a key oncogene in LUAD, promoting tumor progression through both its intrinsic effects on malignant cells and its extrinsic regulation of the TME, notably via TAM recruitment. BST2 expression serves as a robust prognostic marker and a predictor of immunotherapy response. These findings not only nominate BST2 as a promising biomarker for patient stratification but also highlight it as a compelling therapeutic target, opening new avenues for precision medicine in LUAD.

## Author Contributions

**Jianjian Ying and Weiyu Shen**: designed the research. **Keyun Zhu, Mingrong Lin and Chengbin Lin**: performed the research. **Tao Hu, Zhikai Cao, Shuo Huang, Yan Shen and Jing Zeng**: contributed new reagents or analytic tools. **Keyun Zhu, Tao Hu and Zhikai Cao**: writing – review & editing, writing – original draft. **Jinxian He and Jianjian Ying**: revised the manuscript, funding acquisition.

## Funding

This work was supported by the Ningbo Natural Science Foundation (Grants 2023J226 and 2023J224) and the Medical Science and Technology Project of Zhejiang Province (Grant 2024KY297).

## Ethics Statement

Informed consent was not required for the present study, as it did not involve any human subjects or animal experiments.

## Conflicts of Interest

The authors declare no conflicts of interest.

## Supporting Information

Additional supporting information can be found online in the Supporting Information section.

## Supporting information


**Supporting Information 1** Table S1: Primer sequences used for qRT‐PCR experiments. Table S2: The siRNA sequences used for transfection experiments.

**Supporting Information 2** Table S3: The set of genes significantly differentially expressed between LUAD and normal lung tissues.

**Supporting Information 3** Table S4: Module 1 gene set from hdWGCNA analysis.

**Supporting Information 4** Table S5: Potential therapeutic agents targeting BST2‐high phenotypes identified via the Clue.io platform.

**Supporting Information 5** Figure S1: Downregulation of BST2 inhibits LUAD progression in H1975 cells. (A) The BST2 expression levels in lung cancer tissues and normal lung tissues detected by IHC. (B) The cell proliferation of BST2‐deleted H1975 cells detected by MTT assay. (C) Colony formation assay in BST2‐deleted H1975 cells. The cell invasion and migration assay of BST2‐deleted H1975 cells with (D) or without Matrigel (E). (F) Wound healing assays of BST2‐deleted H1975 cells.  ^∗^ *p* < 0.05,  ^∗∗^ *p* < 0.01, and  ^∗∗∗^ *p* < 0.001.

## Data Availability

All data used in this study are retrieved from publicly available sources. The specific repositories are listed in the manuscript/Supporting Information. Further details can be obtained by contacting the corresponding author.
