# Supplementary material for: Single‐Cell Transcriptomics Identifies BST2 as an Oncogenic Driver and Immunotherapy Biomarker in Lung Adenocarcinoma
Source: Mediators Inflamm. 2026 May 6;2026:3777132. doi: 10.1155/mi/3777132 (PMC13147935; doi:10.1155/mi/3777132)
Supplement: Supplementary file 1 — Supporting Information 1 Table S1: Primer sequences used for qRT‐PCR experiments. Table S2: The siRNA sequences used for transfection experiments. [file MI-2026-3777132-s001.docx]

Supplementary file

Table S1. The sequences of primers.

| BST2-Forward Primer | CACACTGTGATGGCCCTAATG |
| --- | --- |
| BST2-Reverse Primer | GTCCGCGATTCTCACGCTT |
| GAPDH-Forward Primer | CAAGGTCATCCATGACAACTTTG |
| GAPDH -Reverse Primer | GTCCACCACCCTGTTGCTGTAG |

Table S2. Oligonucleotides of siRNAs.

| siBST2-1 | sense | GAAUCGCGGACAAGAAGUA |
| --- | --- | --- |
|  | antisense | UACUUCUUGUCCGCGAUUC |
| siBST2-2 | sense | GGAGCGACUGAGAAGAGAA |
|  | antisense | UUCUCUUCUCAGUCGCUCC |
| siBST2-3 | sense | GGAGAGAUCACUACAUUAA |
|  | antisense | UUAAUGUAGUGAUCUCUCC |
