# Supplementary figures and images for: Single‐Cell Transcriptomics Identifies BST2 as an Oncogenic Driver and Immunotherapy Biomarker in Lung Adenocarcinoma
Source: Mediators Inflamm. 2026 May 6;2026:3777132. doi: 10.1155/mi/3777132 (PMC13147935; doi:10.1155/mi/3777132)

**A**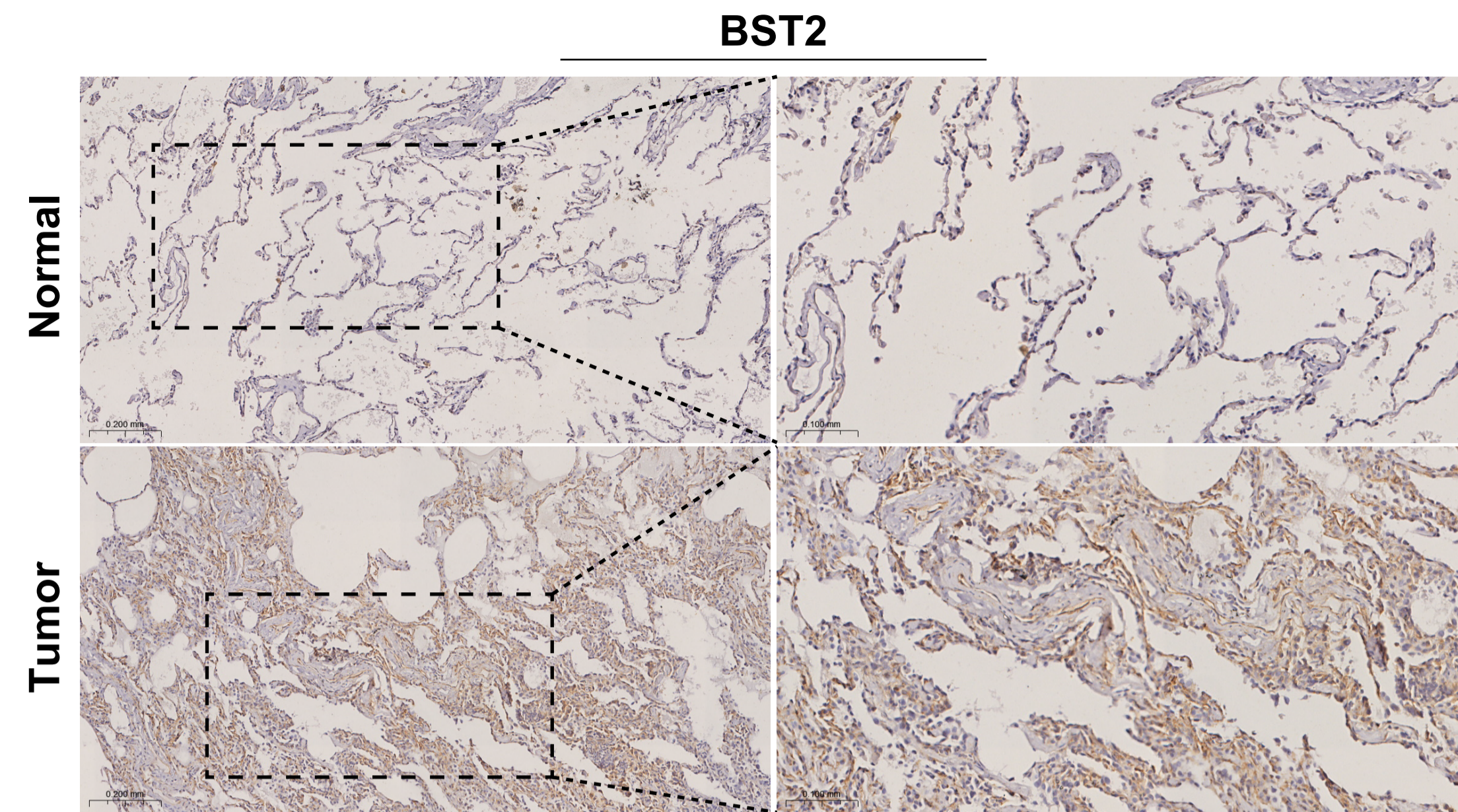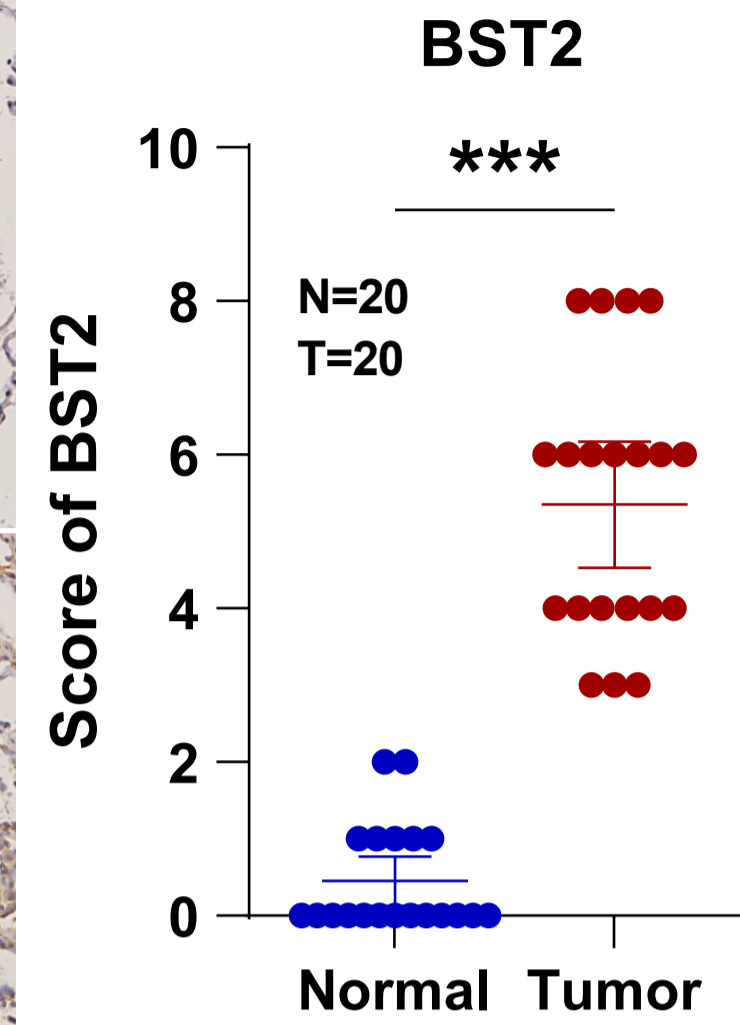**B**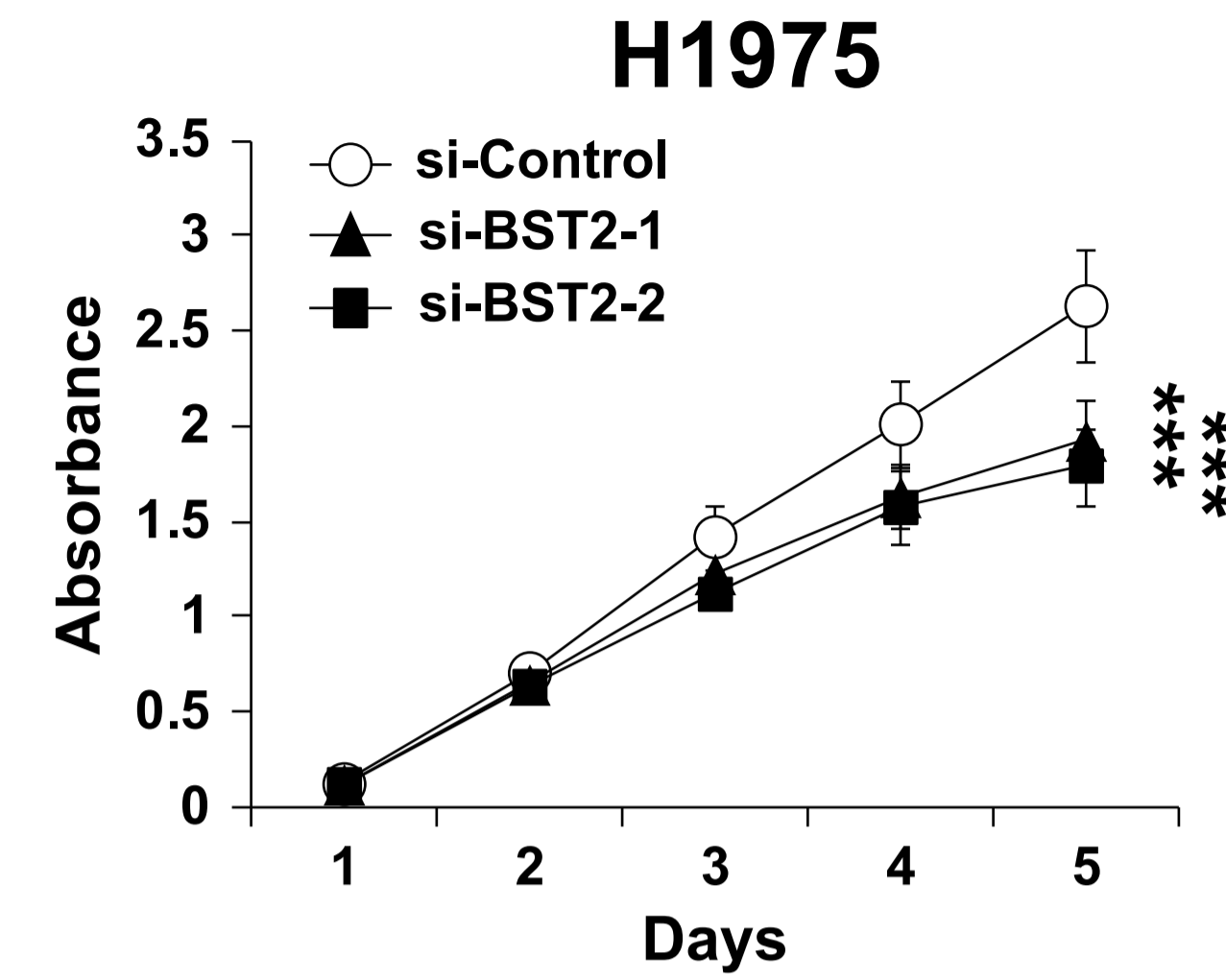**C**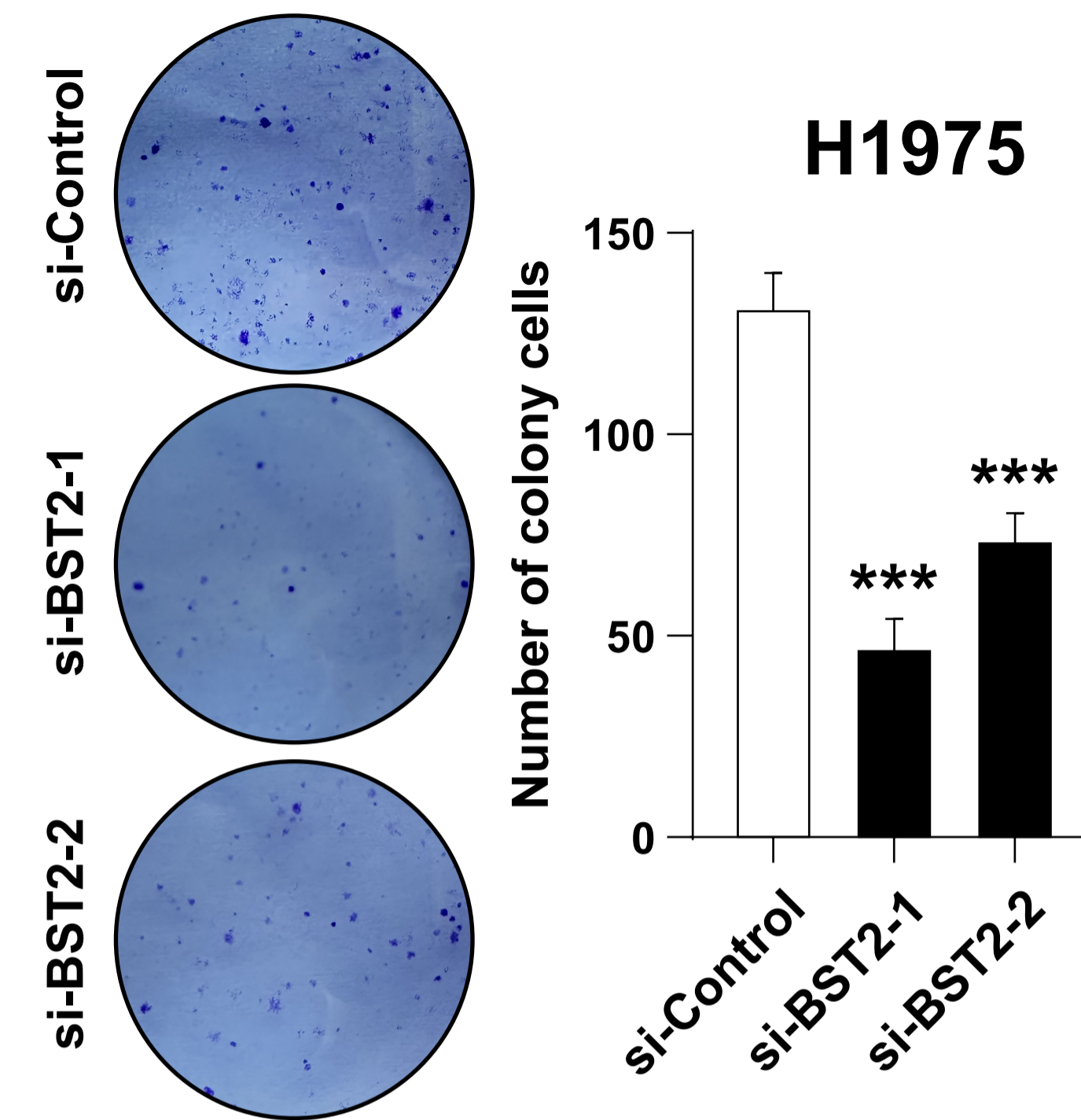**D**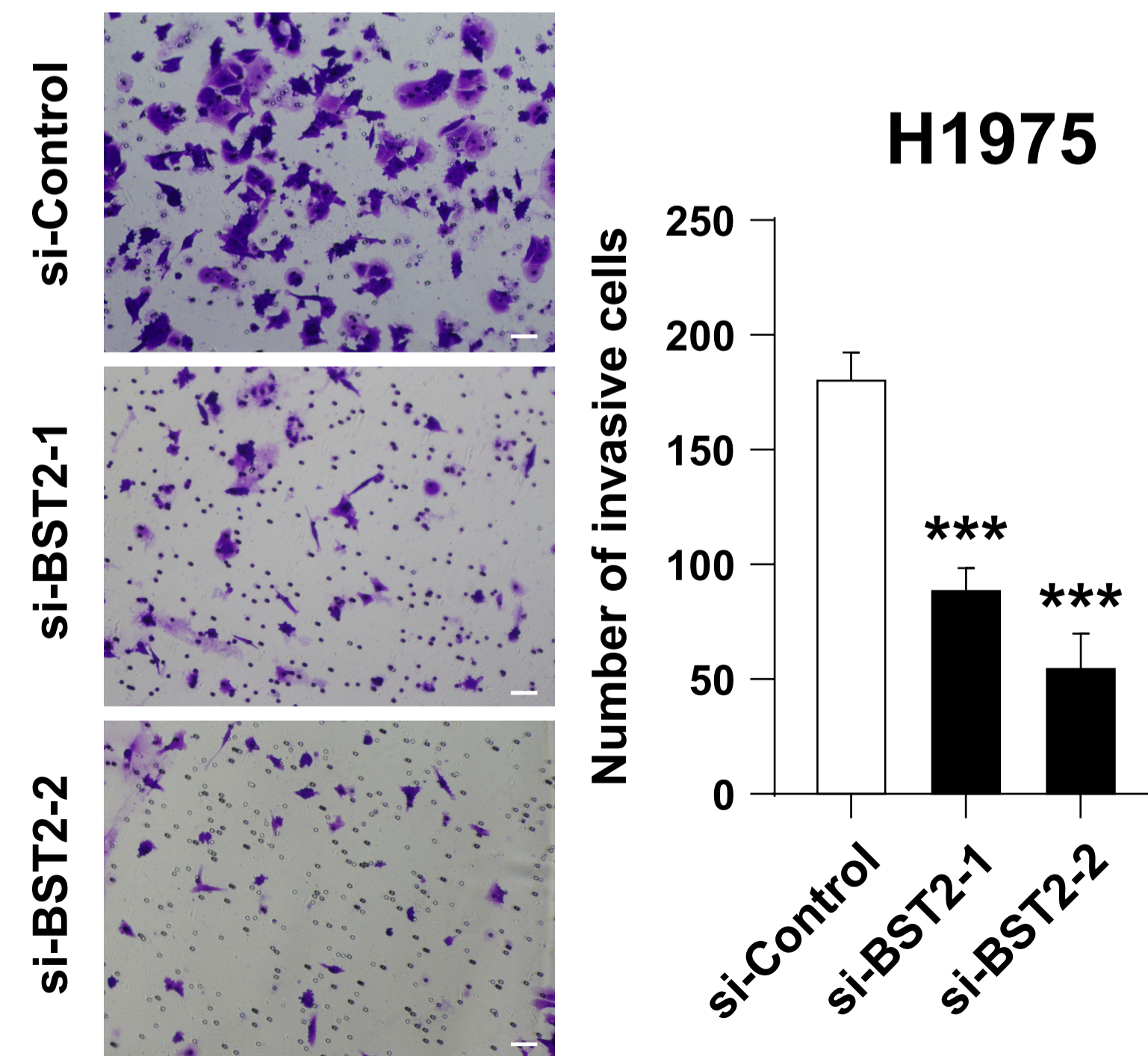**E**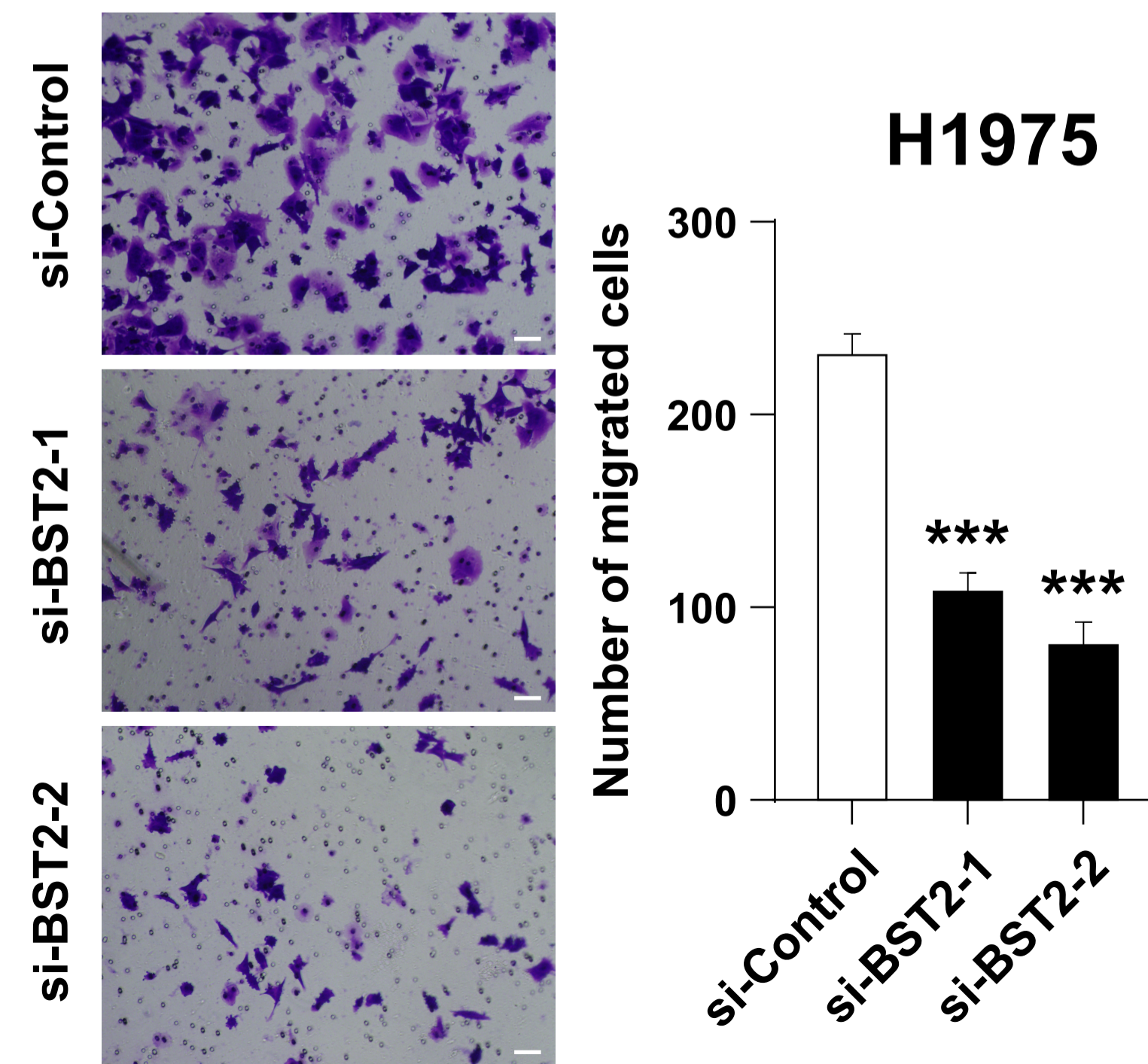**F**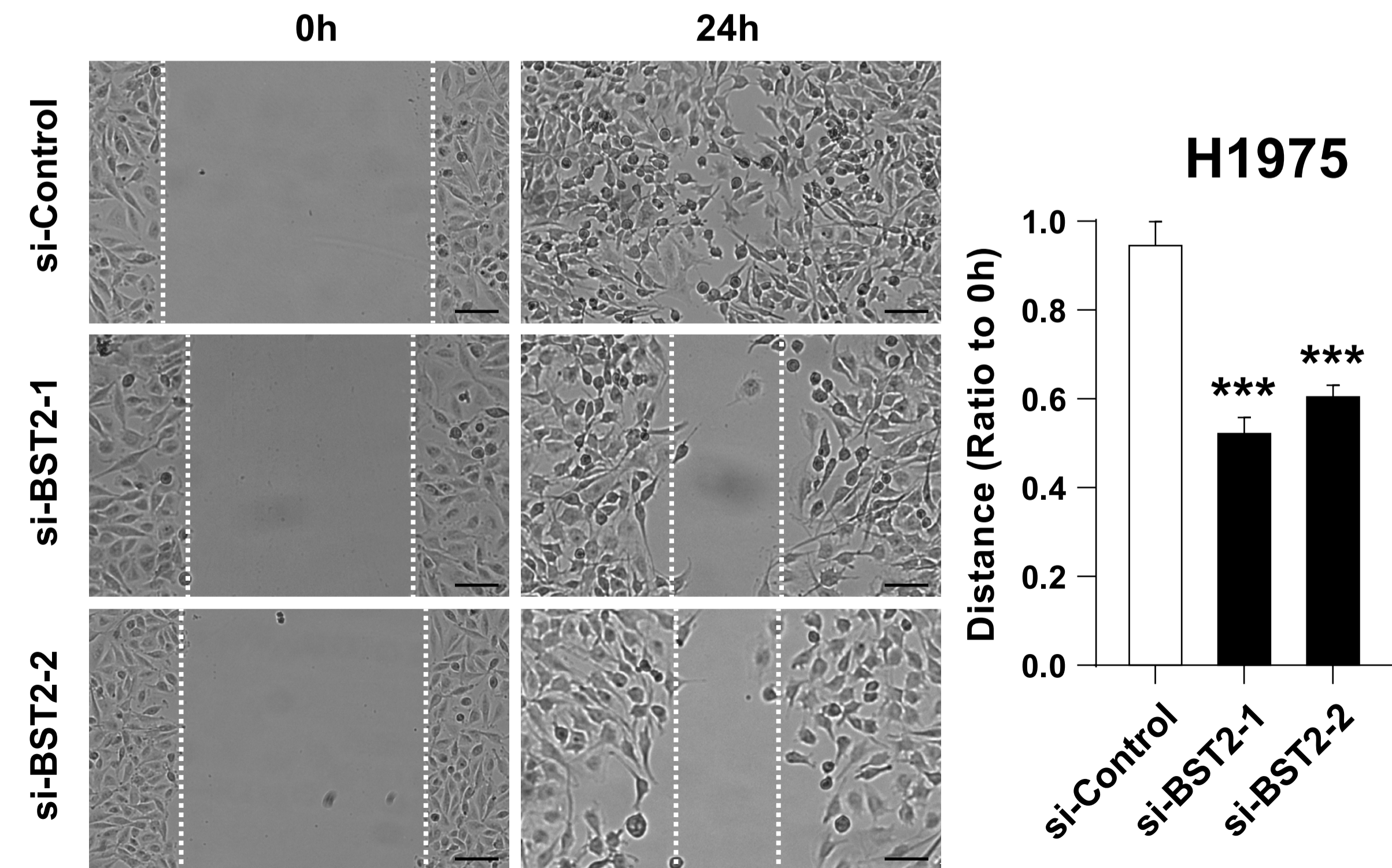

Supplement: Supplementary file 5 — Supporting Information 5 Figure S1: Downregulation of BST2 inhibits LUAD progression in H1975 cells. (A) The BST2 expression levels in lung cancer tissues and normal lung tissues detected by IHC. (B) The cell proliferation of BST2‐deleted H1975 cells detected by MTT assay. (C) Colony formation assay in BST2‐deleted H1975 cells. The cell invasion and migration assay of BST2‐deleted H1975 cells with (D) or without Matrigel (E). (F) Wound healing assays of BST2‐deleted H1975 cells. ∗ p < 0.05, ∗∗ p < 0.01, and ∗∗∗ p < 0.001. [file MI-2026-3777132-s005.pdf]
